# A Survey on the Distribution of Ovothiol and *ovoA* Gene Expression in Different Tissues and Cells: A Comparative Analysis in Sea Urchins and Mussels

**DOI:** 10.3390/md20040268

**Published:** 2022-04-15

**Authors:** Carola Murano, Annalisa Zuccarotto, Serena Leone, Marco Sollitto, Marco Gerdol, Immacolata Castellano, Anna Palumbo

**Affiliations:** 1 Department of Biology and Evolution of Marine Organisms, Stazione Zoologica Anton Dohrn, 80121 Naples, Italy; carola.murano@szn.it (C.M.); annalisa.zuccarotto@szn.it (A.Z.); serena.leone@szn.it (S.L.); immacolata.castellano@szn.it (I.C.); 2 Department of Integrative Marine Ecology, Stazione Zoologica Anton Dohrn, 80121 Naples, Italy; 3 Department of Life Sciences, University of Trieste, 34127 Trieste, Italy; marco.sollitto@phd.units.it (M.S.); mgerdol@units.it (M.G.); 4 Department of Molecular Medicine and Medical Biotechnology, University of Naples Federico II, 80131 Naples, Italy

**Keywords:** ovothiol, sea urchin, mussel, antioxidant, oxidative stress

## Abstract

Ovothiols are histidine-derived thiols produced by a variety of marine invertebrates, protists and bacteria. These compounds, which are among the strongest natural antioxidants, are involved in controlling the cellular redox balance due to their redox exchange with glutathione. Although ovothiols were initially reported as protective agents against environmental stressors, new evidence suggests that they can also act as pheromones and participate in fundamental biological processes such as embryogenesis. To get further insight into the biological roles of ovothiols, we compared ovothiol biosynthesis in the sea urchin *Paracentrotus lividus* and in the mussel *Mytilus galloprovincialis*, the two species that represent the richest sources of these compounds among marine invertebrates. Ovothiol content was measured in different tissues and in the immune cells from both species and the expression levels of *ovoA*, the gene responsible for ovothiol biosynthesis, was inferred from publicly available transcriptomes. A comparative analysis of ovothiol biosynthesis in the two species allowed the identification of the tissues and cells synthesizing the metabolite and highlighted analogies and differences between sea urchins and mussels. By improving our knowledge on the biological roles of ovothiols and pointing out the existence of sustainable natural sources for their isolation, this study provides the basis for future biotechnological investigations on these valuable compounds.

## 1. Introduction

The extreme levels of biodiversity found in marine environments compared with terrestrial habitats has stimulated intense research aimed at the discovery of novel compounds with peculiar biological activities and potential biotechnological applications [1,2]. Among these, ovothiols, π-methyl-5-thiohistidines produced by marine invertebrates, protists and bacteria, have attracted increasing interest for their chemical, biological and pharmacological properties [3,4]. Three forms of ovothiol, termed A, B and C, which differ in the degree of methylation at the Nα of the histidine, are currently known [3,4]. Thanks to their peculiar antioxidant features [5,6], ovothiols were reported to protect sea urchin eggs against the oxidative burst occurring at fertilization and development of embryos against environmental stressors, such as metal ions and toxins [7,8]. Similar defensive functions against environmental stressors have more recently been demonstrated or suggested in other marine organisms, including bony fishes and mussels [9,10]. Moreover, these compounds can protect microalgae from light-induced stress [11,12] and pathogenic parasites from oxidative stress during host infection [13]. Despite these findings, the biological role of ovothiols is likely to be much more complex and not limited to a protective function. Indeed, ovothiols have also been reported to act as pheromones in marine worms [14,15] and to be exploited by cone snails in the context of their peculiar hunting strategy [16]. Recently, ovothiol biosynthesis has also been correlated to some fundamental biological processes during sea urchin embryogenesis, which include cell proliferation, skeleton formation and immune response [17]. Besides its natural involvement in many physiological processes, purified ovothiol A has demonstrated potential as a pharmacological agent, raising interest on the possibility of finding alternative natural sources or to produce it through metabolic engineering. For instance, recent studies have evidenced the therapeutic potential of ovothiol as a regulator of tumor cell growth through the inhibition of human γ-glutamyl transpeptidase [18,19,20] and as an anti-inflammatory agent in both endothelial cells from women affected by gestational diabetes and in vivo murine models of liver fibrosis [21,22,23].

The enzymes responsible for ovothiol biosynthesis are 5-histidylcysteine sulfoxide synthase *ovoA* [24] and beta-lyase OvoB [25]. The evolution of the corresponding genes has been studied in depth, thanks to the increasing availability of genomes of different organisms, from bacteria to metazoa [8,26,27]. However, no specific data has ever been collected concerning the expression pattern of the *ovoA* gene in different tissues of marine invertebrates, not even from the species which represent the major source of the metabolite. To date, sea urchin eggs, which contain ovothiol at millimolar concentrations [28,29], represent the source most commonly used to obtain pure ovothiol A, unmethylated at the amino acidic amino group [4]. However, the accumulation of ovothiol A at high concentrations has also been recently reported in the mantle of female mussels with mature ovaries [10].

To fill the existing knowledge gap about the biological roles of ovothiols and to better understand the distribution and abundance of this metabolite with high biotechnological potential, we performed a detailed investigation on the two richest known biological sources of these compounds, the sea urchin *Paracentrotus lividus* and the mussel *Mytilus galloprovincialis*. The data gathered about the presence of ovothiol A in different tissues and immune cells, complemented with *ovoA* gene expression data resulting from the analysis of publicly available transcriptomic datasets, provided important information about the essential role in different body districts. These results contribute to a deeper understanding of the biological functions of ovothiols in marine organisms, highlighting the existence of abundant exploitable sources of these metabolites, which, in light of their antioxidant and anti-inflammatory properties, possess great potential for biotechnological application.

## 2. Results

The abundance and distribution of ovothiol was investigated by measuring the levels of the metabolite in different tissues of *M. galloprovincialis* and *P. lividus*.

In female mussels, higher levels of ovothiol A were detected in the mantle and gills, followed by the digestive gland. The same trend was observed in males, with the exception of the mantle, where ovothiol levels were significantly lower than females (Table 1).

**Figure 1:**
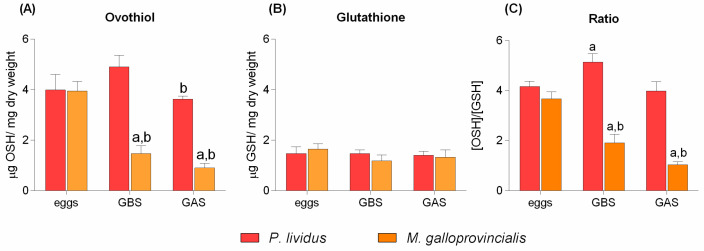
Ovothiol A and glutathione content in *P. lividus* and *M. galloprovincialis* gonads and eggs (**A**–**C**). All data were analyzed by Two-way ANOVA followed by Bonferroni post-test comparing *P. lividus* vs. *M. galloprovincialis*. Bars represent mean ± SD. (**A**) “a” indicates values that are significantly different from the *M. galloprovincialis* eggs (*p*-value < 0.001), “b” is significantly different from the GBS *P. lividus* (*p*-value < 0.05); (**C**) “a” indicates values that are significantly different from the *P. lividus* eggs (*p*-value < 0.05), “b” is significantly different from the *M. galloprovincialis* eggs (*p*-value < 0.001), (*n* = 20). GBS = gonads before spawning; GAS = gonads after spawning.

Ovothiol levels were also measured in the eggs and ovaries of *P. lividus* and *M. galloprovincialis* specimens. The eggs of both species showed comparable amounts of ovothiol, but the gonads of non-spawned sea urchins contained higher levels of the metabolite per mg of dry weight compared to mussels (Figure 1A). After spawning, the amount of ovothiol slightly decreased in sea urchins but the ovaries still contained high levels of the compound (Figure 1A). The levels of glutathione did not significantly change among the different groups (Figure 1B). The molar ratio between ovothiol and glutathione revealed that ovothiol was the predominant antioxidant in the eggs of both species, with a concentration four times higher than glutathione (Figure 1C). In *P. lividus*, ovothiol A was also the predominant thiol in gonads, both before and after spawning (Figure 1C). On the other hand, ovothiol and glutathione were almost equally abundant in the mussel gonads after spawning, as revealed by an ovothiol/glutathione ratio equal to 1.03 ± 0.11 (Figure 1C). This rate was much higher (i.e., 1.91 ± 0.34) in non-spawned gonads, but still lower (about half) than the ratio observed in the eggs.

The higher levels of ovothiol observed in the ovaries of sea urchins, even after the release of mature eggs, suggested that this metabolite was produced in a constitutive manner in the gonads, at least in *P. lividus*. In further support of this idea, the measurements of ovothiol A levels carried out in the gonads from sexually immature specimens collected during the non-breeding season revealed a high level of ovothiol A (3.66 ± 0.02 μg/mg dry weight) and an ovothiol A/glutathione molar ratio equal to 3.8. Histological analyses of these samples show that the ovary was clearly in the recovery stage (Stage I, according to Byrne et al. [30]), confirming that the detection of ovothiol was not dependent on the presence of eggs in the tissue. In fact, just a few previtellogenic oocytes (PO) were visible attached along the acinal wall in the ovary, which was filled with a patchwork of eosinophilic nutritive phagocytes (NP) together with lipid globules (GB) (Figure S1) [31]. To gather further insight into the function of ovothiol in gonadal tissues we determined the levels of ovothiol A and glutathione in male gonads, which accounted for 0.45 ± 0.03 and 0.47 ± 0.07 µg/mg dry weight, respectively.

**Figure 2:**
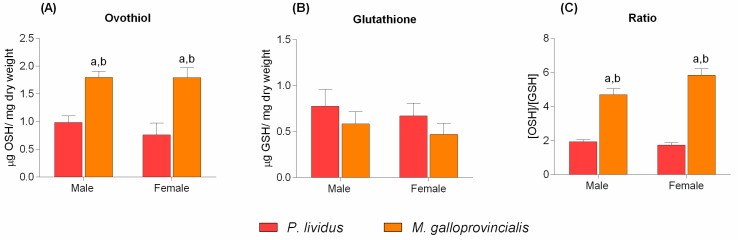
Ovothiol A and glutathione content in *P. lividus* and *M. galloprovincialis* immune cells (**A**–**C**). All data were analyzed by Two-way ANOVA followed by Bonferroni post-test comparing *P. lividus* vs. *M. galloprovincialis.* Bars represent mean ± SD. (**A**,**C**) “a” indicates values that are significantly different from *P. lividus* male (*p*-value < 0.01), “b” is significantly different from *P. lividus* female (*p*-value < 0.05).

Considering that we have previously reported the presence of the *ovoA* transcript in the coelomocytes of *P. lividus* [32], we measured the levels of ovothiol in immune cells of both species. Moderate levels of ovothiol A were detected in *P. lividus* coelomocytes. In this case, the metabolite was more abundant in males than females (0.98 ± 0.12 and 0.76 ± 0.22 µg ovothiol/mg dry weight, respectively), and the amounts detected were double those of glutathione (Figure 2A–C). Interestingly, the hemocytes of *M. galloprovincialis* contained approximately twice as much ovothiol than the sea urchin, with no differences between males and females (Figure 2A).

**Figure 3:**
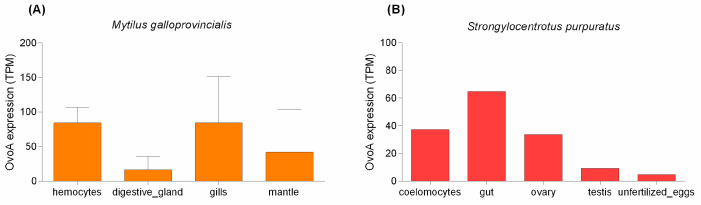
*ovoA* expression in *M. galloprovincialis* and *S. purpuratus*. (**A**) Several RNAseq data were analyzed for *M. galloprovincialis* (Table S1). The bars represent the mean TPM values of *ovoA* in all the run accessions shown in Supplementary Materials (Table S2), aggregated by tissue. (**B**) The bars represent the TPM values of *ovoA* in *S. purpuratus*, obtained from single non-replicated samples.

To further assess the role of ovothiol in the different tissues and cells of *M. galloprovincialis*, we preliminarily investigated the expression of *ovoA* from publicly available transcriptomic datasets. In spite of the different geographical origin of the samples, this approach allowed us to obtain a general overview of the pattern of expression of *ovoA* in this species. High expression was detected in gills and hemocytes, whereas lower TPM values were present in the mantle and digestive gland (Figure 3A). The only two adult tissues of *P. lividus* with available RNAseq datasets are ovary and testis. In both, the observed transcript expression levels were particularly low, accounting for 0.3 and 0.12 TPM, respectively. By contrast, more information, albeit limited to a single biological replicate, is available for *S. purpuratus*, which is known to produce a different ovothiol, ovothiol C, di-methylated at the amino acidic amino group [29]. In this sea urchin species, *ovoA* transcripts were identified as present in significant amounts in the gut, ovary and coelomocytes, whereas lower levels were found in testis and unfertilized eggs (Figure 3B).

## 3. Discussion

In the past few years, a growing interest has been directed worldwide towards marine bioprospecting, looking for pharmaceuticals and novel molecules with biotechnological potential. Although the history of ovothiol dates back to 1980, this molecule is still regarded as one of the most intriguing marine metabolites, due to its intrinsic properties as a potent natural antioxidant and interesting, but still poorly explored, biological functions [4]. The sea urchin *P. lividus* and the mussel *M. galloprovincialis* represent the major known natural sources of ovothiol among marine invertebrates. This study reports, for the first time, a comparative analysis of the distribution of this metabolite in different tissues and cell types in these two species, with the support of transcriptomic data, providing a deeper understanding of the main body districts where ovothiol may play a pivotal role. Since mussel is an edible species and sea urchin eggs are a culinary delicacy, it is very likely that this antioxidant can be absorbed from the diet also by humans, which do not possess the molecular machinery responsible for ovothiols biosynthesis [8,26]. Indeed, ergothioneine, another thiohistidine present in some fungi as well as in beans, can be absorbed from the diet through a protein transporter present on the surface of human cells (OCTN1) [33,34]. The possible uptake of ovothiol through the diet could have important implications for human health, since both ergothioneine and ovothiol are characterized by peculiar antioxidant and anti-inflammatory properties [3,4]. Moreover, since both sea urchin and mussel represent a source of nutrition for other marine species, the bioaccumulation of ovothiol could have important implications also on marine food webs. For example, although bony fishes are not endowed with the genes involved in ovothiol biosynthesis [8,26], they could have acquired membrane transporters to allow uptake from the diet. It is worth mentioning that primary producers, such as microalgae, also produce ovothiol [11,12], which may allow the accumulation of this compound along the food web, moving from phytoplankton to herbivores or filter feeding organisms, up to fish.

One of the key findings of this work is that ovothiol represents the main cellular thiol both in sea urchins and mussels, surpassing in abundance the ubiquitous glutathione. Ovothiols are endowed with unique chemical properties, which mostly derive from the position of the thiol group on the imidazole group of histidine, which determines its low pKa. Therefore, its marked acidic behavior and reductive potential compared to glutathione very likely explain the preference of ovothiol over glutathione in counteracting the severe and stressful conditions typically encountered by marine organisms in their challenging habitat. Indeed, it has been previously suggested that ovothiol is involved in controlling the toxicity of hydrogen peroxide produced by the oxidative burst which occurs at fertilization in *S. purpuratus* eggs [35]. Ovothiol can react with hydrogen peroxide faster than glutathione, to produce ovothiol disulphide which is in turn reduced by glutathione, thus acting as a non-enzymatic glutathione peroxidase system [35].

The extreme abundance of ovothiols in the eggs can be related with its protective role against the oxidative burst occurring at fertilization [7], perhaps also providing protection in embryos when similar conditions are induced by the presence of stressors in the sea water column [8]. Nevertheless, the abundance of ovothiol in *P. lividus* ovaries, at different stages of the reproductive cycle, together with the low levels detected in male gonads, suggests that this molecule may play a role in the maturation and differentiation of female gonads. In fact, sea urchin’s gonads themselves contribute to the high levels of ovothiol measured in this tissue, regardless of the presence of oocytes. On the other hand, while the eggs represent the main source of ovothiol in mussels, the contribution of ovothiol and glutathione appears to be similar in gonads after spawning. Gonadal tissues are well known to have a high intrinsic plasticity, which involves a series of variations between developmental and regressive stages, promoted by reactive oxygen species (ROS) acting as primary or secondary messengers and regulating tissue remodeling and germ cell function [36]. Therefore, it cannot be excluded that ovothiol might represent the main antioxidant constitutively produced in the gonads to protect this tissue from the action of oxygen radicals. Arguably, in a scenario where global changes are becoming more and more predominant and considering the high susceptibility of the gonads to environmental stressors, ovothiol may have acquired a key role in counteracting the oxidative conditions faced by this tissue, modulating gonadal development and function. In this regard, it is plausible that the basal levels of ovothiol and glutathione are influenced by the environmental context of the sampling area of the examined specimens. Specifically, the Gulf of Naples represents a coastal marine area characterized by a high population density, numerous maritime tourist and industrial activities that undoubtedly synergistically favor the concentration of classical and emerging contaminants as well as natural toxins [37,38,39]. The presence of these contaminants is not limited to sea water since, over the years, bioaccumulation can occur at the organismal level as well. Metals and microplastics have in fact been detected in mussels from the Gulf [40,41,42], while in the case of wild sea urchins no data are available except a recent study on the accumulation of anthropogenic microfibers [43]. All these contaminants can alter the redox state in these organisms and consequently induce mechanisms involving the action of ovothiol and/or glutathione, as already suggested in mussels collected in a polluted area or in sea urchin embryos in response to environmental stress conditions [8,10].

Diaz de Cerio and co-authors [10] studied female mussels from Plentzia (Spain), reporting that, during stage 4 of oogenesis, the mantle was the tissue showing the highest concentrations of ovothiol, even superior to the levels obtained in the ovary of sea urchins, which was used as a positive control. In our study, the levels of ovothiol found in the mantle of female mussels were markedly lower than those detected in mussel and sea urchin eggs, and lower than those observed in sea urchin ovary. Moreover, we also revealed that mussel gonads contained lower levels of ovothiol than the eggs, which greatly decreased after spawning. The presence of significant levels of ovothiol in the digestive glands, gills, mantle and hemocytes of the mussel *M. galloprovincialis* is further supported by transcriptomic data, even though the *ovoA* transcript was expressed at higher levels in the gills, mantle and hemocytes, compared with the digestive gland. Interestingly, we observed that gills, among all tissues, showed the highest amounts of ovothiol, both in female and male specimens, with levels only second to unfertilized eggs. As a matter of fact, mussel gills represent the key organ implicated in nutrient uptake, digestion and respiration but also the first effective physical barrier to pathogen invasion and xenobiotics present in the water column [44,45]. In this perspective, the surprising and unique features of ovothiols could provide support for the protection against environmental stressors, establishing a structural mechanism for cellular xenobiotic defense.

Lacking an adaptive immunity, both sea urchins and mussels rely on a cell-mediated immune response by way of free and circulating heterogeneous cells capable of performing phagocytic, cytotoxic or inflammatory activities, to deal with the challenges posed by a broad array of pathogens [46,47]. Not surprisingly, an important outcome of this work is related to the analysis of the ovothiol content in the circulating immune cells of sea urchin and mussel, i.e., coelomocytes and hemocytes, respectively. While no significant difference in terms of ovothiol abundance was detected between male and female individuals in either mussels or sea urchins, the concentration of this metabolite was markedly higher in mussel hemocytes, compared with sea urchin coelomocytes. This trend was confirmed by the expression of *ovoA* transcripts, which showed higher TPM values in mussels. It is worth noting that the ovothiol content in mussel hemocytes almost reached the levels detected in male and female gills, as well as in mantle. These observations corroborate to a greater extent the potential engagement of ovothiol in defense mechanisms and immune functions. Moreover, the expression of *ovoA* in the gut of the sea urchin *S. purpuratus* is consistent with the results we have previously obtained concerning mRNA localization in the gut of *P. lividus* larvae [17]. In fact, the gut is colonized by the microbiome, and the complex interactions in the symbiome are known to be key players in modulating the immune response in sea urchins [48]. In this regard, we have recently observed an increase in *ovoA* transcript levels in sea urchin embryos upon exposure to inflammatory stimuli [17]. In addition to the expression of *ovoA* both in *P. lividus* and *S. purpuratus* coelomocytes, we found significant amounts of the product metabolite in *P. lividus* immune cells. Therefore, the previously demonstrated localization of *ovoA* mRNA in the gut of *P. lividus*, its regulation by inflammation, and the presence of ovothiol in coelomocytes, further highlight the importance of this metabolite in the defense mechanisms of these marine invertebrates.

Although its function is still relatively unknown, ovothiol currently is one of the marine compounds in the spotlight of the scientific community due to its interesting properties for therapeutic and biotechnological purposes. In this context, our study adds a new piece of information to the ovothiol research by providing punctual indications on the amounts and tissue/cell distribution in two common Mediterranean species, *P. lividus* (sea urchins) and *M. galloprovincialis* (mussels). By combining transcriptomic analyses with biochemical techniques, we obtained important indications on the tissues or cells of the sea urchin and mussel in which ovothiol can perform its pivotal roles. As expected, both mussel and sea urchin eggs contain the greatest amount of ovothiol, exceeding 4 µg/mg dry weight, which makes them the ideal source of this metabolite. Nonetheless, sea urchin gonads (before and after spawning) and the mantle/gills of the mussel also provide a significant contribution to the ovothiol content. Therefore, these tissues may represent alternative and viable sources of this metabolite. Considering that our study did not focus on the influence of possible contaminants on the level of ovothiol and the limited availability of transcriptomic data for *P. lividus*, future studies should be devoted to understanding *ovoA* regulation in these Mediterranean species.

## 4. Materials and Method

### 4.1. Animal Collection

Fifty adult specimens of *P. lividus* were collected in the Gulf of Naples from an area not privately owned nor protected, according to the authorization of Marina Mercantile (DPR 1639/68, 09/19/1980, confirmed on 01/10/2000). Sixty adult specimens of *M. galloprovincialis* were purchased from a commercial shellfish farm (Bacoli, Napoli, Italy). Although no authorization is required for sea urchins and mussels, all animal procedures were in compliance with the guidelines of the European Union (directive 2010/63/EU and following D. Lgs. 4/03/2014 n.26) on the protection of animals used for scientific purposes by reducing the minimum the number of specimens used and any pain or stress on animals.

### 4.2. Tissue and Cells Sampling

*P. lividus* eggs were collected after injection of sea urchin with a 0.5 M KCl solution through the peribuccal membrane, while spawning in *M. galloprovincialis* was performed leaving specimens in beakers with natural seawater (NSW). Eggs were then collected by centrifugation at 1000× *g* for 10 min at 4 °C. Consequently, the resulting ovaries represented the gonads after spawning (GAS) without the mature eggs. Conversely, gonads not spawned (containing eggs) represented the gonads before spawning (GBS) and were obtained directly by dissecting mature specimens, avoiding the spawning process. All the tissues and eggs were stored at −20 °C until further analysis. The coelomic fluid in sea urchin was withdrawn through a puncture (needle 26 gauge) in the peristomial membrane using a sterile syringe (1 mL) pre-loaded with an anticoagulant solution as previously described in Murano et al. [49]. Instead, mussel hemolymph was withdrawn from the mussel’s adductor muscle using a sterile syringe (1 mL) pre-loaded with a buffer solution according to Liberatori et al. [50]. The coelomocytes and hemocytes were then collected by centrifugation at 600× *g* for 20 min at 4 °C and stored at −20 °C until analysis. For sea urchins, ovaries from specimens sampled in a non-breeding period as well as gonads from males sampled in the reproductive season were collected, weighed and stored at −20 °C until further processing. The same procedure was performed for the tissues (digestive gland, gills and mantle) from *M. galloprovincialis* specimens.

### 4.3. Ovothiol Determination

Ovothiol A and glutathione were quantified by RP-HPLC analysis of their 4-bromomethyl-7-methoxycoumarin (BMC) derivatives, prepared according to a modification of the procedure described in Milito et al. [11]. Briefly, 10 mg of freeze-dried samples of the different tissues were resuspended in 20 µL of water. Samples were lysed with 90 µL of HClO_4_ 0.75 M: AcCN 2:1 and spiked with 10 µL of 1 mM N-Acetyl-Cysteine (NAC) as internal standard. After extensive vortexing, insoluble debris was removed by centrifugation (5 min, 16,000× *g*) and excess HClO_4_ was removed from 100 µL of supernatant by precipitation with 15 µL of K_2_CO_3_ 2M. After removal of potassium perchlorate by centrifugation, 100 µL of the supernatant were basified by addition of 10 µL of 50 mM Li_2_CO_3_. The thiols in solution were reduced with 3 µL of 200 mM DTT, incubating 5 min before the addition of 25 µL of BMC 20 mM in DMSO. The reaction was allowed to proceed for 30 min in the dark and then stopped by the addition of 10 µL of 10% formic acid. Samples were extensively centrifuged to remove excess BMC before analysis on an Agilent 1260 Infinity II system equipped with a Poroshell 120 EC-C18 column (4 µm, 150 × 4.6 mm, Agilent) and UV detection at 330 nm. The mobile phase was a gradient of AcCN containing 0.1% formic acid (B) in 0.1% formic acid (A) at a flow rate of 0.8 mL min^−1^ as follows: 0.0–2.0 min, 2% B; 2.0–6.0 min, 9% B; 6.0–12.5 min, 6–46% B; 12.5–14.0 min, 46–90% B; 14.0–17.0 min, 90% B; 17.0–19.0 min, 90–2% B. Each injection was followed by a 5-min equilibration at 2% B. Peak identity was confirmed by comparison with authentic standards.

### 4.4. Histological Analysis

Briefly, gonads were fixed in Bouin’s fixative solution followed by the paraffin embedding process [30]. Afterward, gonads embedded in paraffin wax were sectioned in 7 μm thin sections using a Paraffin Rotary Microtome RM2245 (Leica Microsystems, Inc., Buffalo Grove, IL, USA). The sections were then stained with hematoxylin and eosin and mounted between a glass slide and cover slip with synthetic resin according to Machado et al. [51].

### 4.5. In Silico Analysis of ovoA Expression in Sea Urchin and Mussel Tissues and Cells

For the transcriptomic analysis, raw RNAseq data used in this study were downloaded from the NCBI Sequence Read Archive (SRA) database (Supplementary Material, Tables S1 and S2). Raw reads were quality assessed, trimmed to remove sequencing adapters and low-quality bases using fastp 0.20.0 [52]. Afterwards, mitochondrial and ribosomal sequences were filtered out using, respectively, Bowtie2 [53] and SortMeRNA 2.1b [54]. Filtered reads of *P. lividus* were de novo assembled to create a reference transcriptome, using Oyster River protocol 2.3.3 pipeline [55]. For *S. purpuratus* and *M. galloprovincialis*, resulting reads were mapped against the reference transcriptomes through Salmon 1.5.2 [56], and the transcript per million (TPM) for each transcript in different tissues was calculated. The assessment of the presence of bona fide *ovoA* sequences was carefully evaluated by BLASTn with known *ovoA* sequences, and through the functional annotation of associated and conserved protein domains, via Interproscan analysis [57].

### 4.6. Statistical Analysis

The data on ovothiol/glutathione content in *P. lividus* and *M. galloprovincialis* were analyzed by two-way analysis of variance ANOVA (*p* < 0.05) followed by Bonferroni’s multiple comparisons test. Data are presented as mean ± SD and statistics were performed using GraphPad Prism version 7.00 for Windows.

## Figures and Tables

**Table 1:** Ovothiol A levels in *M. galloprovincialis*.

	μg Ovothiol/mg Dry Weight	
Tissue	Female	Male
Digestive gland	1.4 ± 0.04	1.0 ± 0.02
Gills	2.0 ± 0.05	2.5 ± 0.10
Mantle	2.3 ± 0.22	0.29 ± 0.01

## Data Availability

All data are contained within this article and Supplementary Materials.

## Supplementary Materials

The following supporting information can be downloaded at: https://www.mdpi.com/article/10.3390/md20040268/s1, Figure S1: Histological analysis; Table S1: RNAseq raw-data for *M. galloprovincialis*; Table S2: RNAseq raw-data for *P. lividus* and *S. purpuratus*.

## References

[1] Blunt J.W., Carroll A.R., Copp B.R., Davis R.A., Keyzers R.A., Prinsep M.R. (2018). Marine Natural Products. Nat. Prod. Rep..

[2] Carroll A.R., Copp B.R., Davis R.A., Keyzers R.A., Prinsep M.R. (2020). Marine Natural Products. Nat. Prod. Rep..

[3] Castellano I., Seebeck F.P. (2018). On Ovothiol Biosynthesis and Biological Roles: From Life in the Ocean to Therapeutic Potential. Nat. Prod. Rep..

[4] Palumbo A., Castellano I., Napolitano A. (2018). Ovothiol: A Potent Natural Antioxidant from Marine Organisms. Blue Biotechnology.

[5] Osik N.A., Zelentsova E.A., Tsentalovich Y.P. (2021). Kinetic Studies of Antioxidant Properties of Ovothiol A. Antioxidants.

[6] Marjanovic B., Simic M.G., Jovanovic S.V. (1995). Heterocyclic Thiols as Antioxidants: Why Ovothiol C Is a Better Antioxidant than Ergothioneine. Free Radic. Biol. Med..

[7] Shapiro B.M. (1991). The Control of Oxidant Stress at Fertilization. Science.

[8] Castellano I., Migliaccio O., D’Aniello S., Merlino A., Napolitano A., Palumbo A. (2016). Shedding Light on Ovothiol Biosynthesis in Marine Metazoans. Sci. Rep..

[9] Yanshole V.V., Yanshole L.V., Zelentsova E.A., Tsentalovich Y.P. (2019). Ovothiol A Is the Main Antioxidant in Fish Lens. Metabolites.

[10] Diaz de Cerio O., Reina L., Squatrito V., Etxebarria N., Gonzalez-Gaya B., Cancio I. (2020). Gametogenesis-Related Fluctuations in Ovothiol Levels in the Mantle of Mussels from Different Estuaries: Fighting Oxidative Stress for Spawning in Polluted Waters. Biomolecules.

[11] Milito A., Castellano I., Burn R., Seebeck F.P., Brunet C., Palumbo A. (2020). First Evidence of Ovothiol Biosynthesis in Marine Diatoms. Free. Radic. Biol. Med..

[12] Milito A., Orefice I., Smerilli A., Castellano I., Napolitano A., Brunet C., Palumbo A. (2020). Insights into the Light Response of Skeletonema Marinoi: Involvement of Ovothiol. Marine Drugs.

[13] Ariyanayagam M.R., Fairlamb A.H. (2001). Ovothiol and Trypanothione as Antioxidants in Trypanosomatids. Mol. Biochem. Parasitol..

[14] Röhl I., Schneider B., Schmidt B., Zeeck E. (1999). ʟ-Ovothiol A: The Egg Release Pheromone of the Marine Polychaete Platynereis Dumerilii: Annelida: Polychaeta. Z. Für Nat. C.

[15] Breithaupt T., Hardege J.D., Brönmark C., Hansson L.-A. (2012). Pheromones mediating sex and dominance in aquatic animals in Chemical ecology in aquatic systems. Chemical Ecology in Aquatic Systems.

[16] Torres J.P., Lin Z., Watkins M., Salcedo P.F., Baskin R.P., Elhabian S., Safavi-Hemami H., Taylor D., Tun J., Concepcion G.P. (2021). Small-Molecule Mimicry Hunting Strategy in the Imperial Cone Snail Conus Imperialis. Sci. Adv..

[17] Milito A., Cocurullo M., Columbro A., Nonnis S., Tedeschi G., Castellano I., Arnone M.I., Palumbo A. (2022). Ovothiol Ensures the Correct Developmental Programme of the Sea Urchin *Paracentrotus Lividus* Embryo. Open Biol..

[18] Russo G., Russo M., Castellano I., Napolitano A., Palumbo A. (2014). Ovothiol Isolated from Sea Urchin Oocytes Induces Autophagy in the Hep-G2 Cell Line. Mar. Drugs.

[19] Brancaccio M., Russo M., Masullo M., Palumbo A., Russo G.L., Castellano I. (2019). Sulfur-Containing Histidine Compounds Inhibit γ-Glutamyl Transpeptidase Activity in Human Cancer Cells. J. Biol. Chem..

[20] Milito A., Brancaccio M., Lisurek M., Masullo M., Palumbo A., Castellano I. (2019). Probing the Interactions of Sulfur-Containing Histidine Compounds with Human Gamma-Glutamyl Transpeptidase. Mar. Drugs.

[21] Brancaccio M., D’Argenio G., Lembo V., Palumbo A., Castellano I. (2018). Antifibrotic Effect of Marine Ovothiol in an *In Vivo* Model of Liver Fibrosis. Oxidative Med. Cell. Longev..

[22] Castellano I., Di Tomo P., Di Pietro N., Mandatori D., Pipino C., Formoso G., Napolitano A., Palumbo A., Pandolfi A. (2018). Anti-Inflammatory Activity of Marine Ovothiol A in an In Vitro Model of Endothelial Dysfunction Induced by Hyperglycemia. Oxidative Med. Cell. Longev..

[23] Milito A., Brancaccio M., D’Argenio G., Castellano I. (2019). Natural Sulfur-Containing Compounds: An Alternative Therapeutic Strategy against Liver Fibrosis. Cells.

[24] Braunshausen A., Seebeck F.P. (2011). Identification and Characterization of the First Ovothiol Biosynthetic Enzyme. J. Am. Chem. Soc..

[25] Naowarojna N., Huang P., Cai Y., Song H., Wu L., Cheng R., Li Y., Wang S., Lyu H., Zhang L. (2018). In Vitro Reconstitution of the Remaining Steps in Ovothiol A Biosynthesis: C–S Lyase and Methyltransferase Reactions. Org. Lett..

[26] Gerdol M., Sollitto M., Pallavicini A., Castellano I. (2019). The Complex Evolutionary History of Sulfoxide Synthase in Ovothiol Biosynthesis. Proc. R. Soc. B..

[27] Brancaccio M., Tangherlini M., Danovaro R., Castellano I. (2021). Metabolic Adaptations to Marine Environments: Molecular Diversity and Evolution of Ovothiol Biosynthesis in Bacteria. Genome Biol. Evol..

[28] Palumbo A., Misuraca G., d’Ischia M., Donaudy F., Prota G. (1984). Isolation and Distribution of 1-Methyl-5-Thiol-l-Histidine Disulphide and a Related Metabolite in Eggs from Echinoderms. Comp. Biochem. Physiol. Part B Comp. Biochem..

[29] Turner E., Klevit R., Hopkins P.B., Shapiro B.M. (1986). Ovothiol: A Novel Thiohistidine Compound from Sea Urchin Eggs That Confers NAD(P)H-O_2_ Oxidoreductase Activity on Ovoperoxidase. J. Biol. Chem..

[30] Byrne M. (1990). Annual Reproductive Cycles of the Commercial Sea Urchin *Paracentrotus Lividus* from an Exposed Intertidal and a Sheltered Subtidal Habitat on the West Coast of Ireland. Mar. Biol..

[31] Santos P.M., Albano P., Raposo A., Ferreira S.M.F., Costa J.L., Pombo A. (2020). The Effect of Temperature on Somatic and Gonadal Development of the Sea Urchin *Paracentrotus Lividus* (Lamarck, 1816). Aquaculture.

[32] Milito A., Murano C., Castellano I., Romano G., Palumbo A. (2020). Antioxidant and Immune Response of the Sea Urchin *Paracentrotus Lividus* to Different Re-Suspension Patterns of Highly Polluted Marine Sediments. Mar. Environ. Res..

[33] Grundemann D., Harlfinger S., Golz S., Geerts A., Lazar A., Berkels R., Jung N., Rubbert A., Schomig E. (2005). Discovery of the Ergothioneine Transporter. Proc. Natl. Acad. Sci. USA.

[34] Halliwell B., Cheach I.K., Tang R.M.Y. (2018). Ergothioneine—A diet-derived antioxidant with therapeuticpotential. Febs Lett..

[35] Shapiro B.M., Turner E. (1988). Oxidative Stress and the Role of Novel Thiol Compounds at Fertilization. Biofactors.

[36] Riley J.C.M., Behrman H.R. (1991). Oxygen Radicals and Reactive Oxygen Species in Reproduction. Proc. Soc. Exp. Biol. Med..

[37] Cianelli D., Uttieri M., Buonocore B., Falco P., Zambardino G., Zambianchi E., William G.S. (2012). Dynamics of a very special Mediterranean coastal area: The Gulf of Naples. Mediterranean Ecosystems: Dynamics, Management & Conservation.

[38] Montuori P., Lama P., Aurino S., Naviglio D., Triassi M. (2013). Metals Loads into the Mediterranean Sea: Estimate of Sarno River Inputs and Ecological Risk. Ecotoxicology.

[39] Tornero V., Ribera d’Alcalà M. (2014). Contamination by Hazardous Substances in the Gulf of Naples and Nearby Coastal Areas: A Review of Sources, Environmental Levels and Potential Impacts in the MSFD Perspective. Sci. Total Environ..

[40] Fasano E., Arnese A., Esposito F., Albano L., Masucci A., Capelli C., Cirillo T., Nardone A. (2018). Evaluation of the impact of anthropogenic activities on arsenic, cadmium, chromium, mercury, lead, and polycyclic aromatic hydrocarbon levels in seafood from the Gulf of Naples, Italy. J. Environ. Sci. Health Part A.

[41] Perugini M., Visciano P., Manera M., Turno G., Lucisano A., Amorena M. (2007). Polycyclic Aromatic Hydrocarbons in Marine Organisms from the Gulf of Naples. Tyrrhenian Sea. J. Agric. Food Chem..

[42] Mercogliano R., Santonicola S., De Felice A., Anastasio A., Murru N., Ferrante M.C., Cortesi M.L. (2016). Occurrence and Distribution of Polycyclic Aromatic Hydrocarbons in Mussels from the Gulf of Naples, Tyrrhenian Sea, Italy. Mar. Pollut. Bull..

[43] Murano C., Vaccari L., Casotti R., Corsi I., Palumbo A. (2022). Occurrence of microfibres in wild specimens of adult sea urchin *Paracentrotus lividus* (Lamarck, 1816) from a coastal area of the central Mediterranean Sea. Mar. Pollut. Bull..

[44] Mitta G., Vandenbulcke F., Noel T., Romestand B., Beauvillain J.C., Salzet M., Roch P. (2000). Differential Distribution and Defence Involvement of Antimicrobial Peptides in Mussel. J. Cell Sci..

[45] Gómez-Mendikute A., Elizondo M., Venier P., Cajaraville M.P. (2005). Characterization of Mussel Gill Cells in Vivo and in Vitro. Cell Tissue Res..

[46] Smith L.C., Arizza V., Barela Hudgell M.A., Barone G., Bodnar A.G., Buckley K.M., Cunsolo V., Dheilly N.M., Franchi N., Fugmann S.D., Cooper E.L. (2018). Echinodermata: The Complex Immune System in Echinoderms. Advances in Comparative Immunology.

[47] Canesi L., Ciacci C., Balbi T. (2016). Invertebrate Models for Investigating the Impact of Nanomaterials on Innate Immunity: The Example of the Marine Mussel *Mytilus* spp.. Curr. Bionanotechnol..

[48] Buckley K.M., Rast J.P. (2019). Immune activity at the gut epithelium in the larval sea urchin. Cell Tissue Res..

[49] Murano C., Bergami E., Liberatori G., Palumbo A., Corsi I. (2021). Interplay Between Nanoplastics and the Immune System of the Mediterranean Sea Urchin *Paracentrotus Lividus*. Front. Mar. Sci..

[50] Liberatori G., Grassi G., Guidi P., Bernardeschi M., Fiorati A., Scarcelli V., Genovese M., Faleri C., Protano G., Frenzilli G. (2020). Effect-Based Approach to Assess Nanostructured Cellulose Sponge Removal Efficacy of Zinc Ions from Seawater to Prevent Ecological Risks. Nanomaterials.

[51] Machado I., Moura P., Pereira F., Vasconcelos P., Gaspar M.B. (2019). Reproductive Cycle of the Commercially Harvested Sea Urchin (*Paracentrotus Lividus*) along the Western Coast of Portugal. Invertebr. Biol..

[52] Chen S., Zhou Y., Chen Y., Gu J. (2018). Fastp: An Ultra-Fast All-in-One FASTQ Preprocessor. Bioinformatics.

[53] Langmead B., Salzberg S.L. (2012). Fast Gapped-Read Alignment with Bowtie 2. Nat. Methods.

[54] Kopylova E., Noé L., Touzet H. (2012). SortMeRNA: Fast and Accurate Filtering of Ribosomal RNAs in Metatranscriptomic Data. Bioinformatics.

[55] MacManes M.D. (2018). The Oyster River Protocol: A Multi-Assembler and Kmer Approach for de Novo Transcriptome Assembly. PeerJ.

[56] Patro R., Duggal G., Love M.I., Irizarry R.A., Kingsford C. (2017). Salmon Provides Fast and Bias-Aware Quantification of Transcript Expression. Nat. Methods.

[57] Jones P., Binns D., Chang H.-Y., Fraser M., Li W., McAnulla C., McWilliam H., Maslen J., Mitchell A., Nuka G. (2014). InterProScan 5: Genome-Scale Protein Function Classification. Bioinformatics.

