# Supplementary material for: A Survey on the Distribution of Ovothiol and ovoA Gene Expression in Different Tissues and Cells: A Comparative Analysis in Sea Urchins and Mussels
Source: Mar Drugs. 2022 Apr 15;20(4):268. doi: 10.3390/md20040268 (PMC9029387; doi:10.3390/md20040268)
Supplement: Supplementary file 1 [file marinedrugs-20-00268-s001.zip › marinedrugs-1624707-supplementary.pdf]

Supplementary material

A survey on the distribution of ovothiol and *ovoA* gene expres-sion in different tissues and cells: a comparative analysis in sea urchin and mussels

Carola Murano[1,2], Annalisa Zuccarotto[1], Serena Leone[1], Marco Sollitto[3], Marco Gerdol[3], Immacolata Castellano[1,4,] and Anna Palumbo [1,*]

[1] Department of Biology and Evolution of Marine Organisms, Stazione Zoologica Anton Dohrn, Naples, Italy;
[2] Department of Integrative Marine Ecology, Stazione Zoologica Anton Dohrn; Naples, Italy;
[3] Department of Life Sciences, University of Trieste, Trieste, Italy;
[4] Department of Molecular Medicine and Medical Biotechnology, University of Naples Federico II, Italy

* Correspondence: A.P. anna.palumbo@szn.it

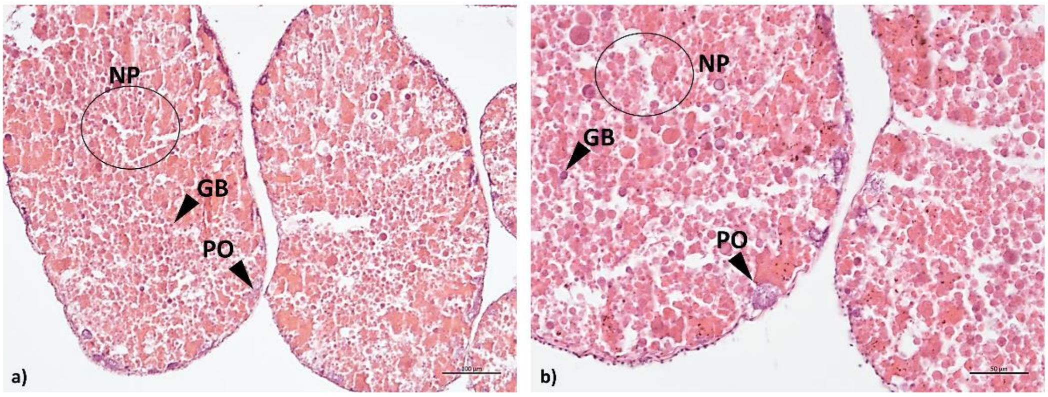

Figure S1. Histology of *P. lividus* ovaries. (a-b) Cross-section through the acinus of the ovary in the recovery stage (Stage I, according to Byrne (1990)). PO, previtellogenic oocytes; NP, nutritive phagocytes; GB, nutritive phagocytes with nutritive droplets. Scale bars: (a)= 100 µm and (b)= 50 µm.

Table S1. RNAseq raw-data (NCBI Sequence Read Archive database) found for *M. galloprovincialis*

**Bioproject**	**SRA accession**	**Species**	**Tissue**
PRJNA88481	SRR442036	*Mytilus galloprovincialis*	digestive gland
PRJNA230138	SRR1046119	*Mytilus galloprovincialis*	gills
PRJNA230138	SRR1042397	*Mytilus galloprovincialis*	hemocytes
PRJNA230138	SRR1045900	*Mytilus galloprovincialis*	hemocytes
PRJNA230138	SRR1046115	*Mytilus galloprovincialis*	mantle
PRJNA230138	SRR1046116	*Mytilus galloprovincialis*	mantle
PRJNA638821	SRR11996464	*Mytilus galloprovincialis*	gills
PRJNA638821	SRR11996686	*Mytilus galloprovincialis*	gills
PRJNA638821	SRR11996723	*Mytilus galloprovincialis*	gills

PRJNA611904	SRR11285774	*Mytilus galloprovincialis*	digestive gland
PRJNA611904	SRR11285775	*Mytilus galloprovincialis*	digestive gland
**Bioproject**	**SRA accession**	**Species**	**Tissue**
PRJNA611904	SRR11285777	*Mytilus galloprovincialis*	digestive gland
PRJNA611904	SRR11285778	*Mytilus galloprovincialis*	digestive gland
PRJNA560413	SRR9984957	*Mytilus galloprovincialis*	mantle
PRJNA560413	SRR9984958	*Mytilus galloprovincialis*	mantle
PRJNA560413	SRR9984959	*Mytilus galloprovincialis*	mantle
PRJNA560413	SRR9984960	*Mytilus galloprovincialis*	mantle
PRJNA560413	SRR9984961	*Mytilus galloprovincialis*	mantle
PRJNA560413	SRR9984962	*Mytilus galloprovincialis*	mantle
PRJNA560413	SRR9984963	*Mytilus galloprovincialis*	mantle
PRJNA560413	SRR9984964	*Mytilus galloprovincialis*	mantle
PRJNA560413	SRR9984965	*Mytilus galloprovincialis*	mantle
PRJNA560413	SRR9984966	*Mytilus galloprovincialis*	mantle
PRJNA560413	SRR9984967	*Mytilus galloprovincialis*	mantle
PRJNA560413	SRR9984968	*Mytilus galloprovincialis*	mantle
PRJNA560413	SRR9984975	*Mytilus galloprovincialis*	mantle
PRJNA560413	SRR9984976	*Mytilus galloprovincialis*	mantle
PRJNA560413	SRR9984977	*Mytilus galloprovincialis*	mantle
PRJNA560413	SRR9984978	*Mytilus galloprovincialis*	mantle
PRJNA560413	SRR9984979	*Mytilus galloprovincialis*	mantle
PRJNA560413	SRR9984980	*Mytilus galloprovincialis*	mantle
PRJNA560413	SRR9984981	*Mytilus galloprovincialis*	mantle
PRJNA560413	SRR9984982	*Mytilus galloprovincialis*	mantle
PRJNA560413	SRR9984983	*Mytilus galloprovincialis*	mantle
PRJNA560413	SRR9984984	*Mytilus galloprovincialis*	mantle
PRJNA560413	SRR9984985	*Mytilus galloprovincialis*	mantle
PRJNA560413	SRR9984986	*Mytilus galloprovincialis*	mantle
PRJNA560413	SRR9984987	*Mytilus galloprovincialis*	mantle
PRJNA560413	SRR9984988	*Mytilus galloprovincialis*	mantle
PRJNA560413	SRR9984989	*Mytilus galloprovincialis*	mantle
PRJNA560413	SRR9984991	*Mytilus galloprovincialis*	mantle
PRJNA560413	SRR9984992	*Mytilus galloprovincialis*	mantle
PRJNA560413	SRR9984993	*Mytilus galloprovincialis*	mantle
PRJNA560413	SRR9984994	*Mytilus galloprovincialis*	mantle
PRJNA560413	SRR9984995	*Mytilus galloprovincialis*	mantle
PRJNA560413	SRR9984996	*Mytilus galloprovincialis*	mantle
PRJNA561723	SRR10015745	*Mytilus galloprovincialis*	digestive gland
PRJNA561723	SRR10015746	*Mytilus galloprovincialis*	digestive gland
PRJNA561723	SRR10015747	*Mytilus galloprovincialis*	digestive gland
PRJNA561723	SRR10015748	*Mytilus galloprovincialis*	digestive gland
PRJNA561723	SRR10015749	*Mytilus galloprovincialis*	digestive gland
PRJNA561723	SRR10015750	*Mytilus galloprovincialis*	digestive gland
PRJNA561723	SRR10015751	*Mytilus galloprovincialis*	digestive gland
PRJNA561723	SRR10015757	*Mytilus galloprovincialis*	digestive gland
PRJNA561723	SRR10015766	*Mytilus galloprovincialis*	digestive gland

PRJNA561723	SRR10015772	*Mytilus galloprovincialis*	digestive gland
PRJNA561723	SRR10015783	*Mytilus galloprovincialis*	digestive gland
**Bioproject**	**SRA accession**	**Species**	**Tissue**
PRJNA451093	SRR7044533	*Mytilus galloprovincialis*	mantle
PRJNA526432	SRR8707268	*Mytilus galloprovincialis*	gills
PRJNA484309	SRR7640805	*Mytilus galloprovincialis*	gills
PRJNA484309	SRR7640807	*Mytilus galloprovincialis*	gills
PRJNA484309	SRR7640808	*Mytilus galloprovincialis*	gills
PRJNA484309	SRR7640811	*Mytilus galloprovincialis*	gills
PRJNA484309	SRR7640812	*Mytilus galloprovincialis*	gills
PRJNA484309	SRR7640813	*Mytilus galloprovincialis*	gills
PRJNA484309	SRR7640814	*Mytilus galloprovincialis*	gills
PRJNA484309	SRR7640818	*Mytilus galloprovincialis*	gills
PRJNA484309	SRR7640820	*Mytilus galloprovincialis*	gills
PRJNA484309	SRR7640821	*Mytilus galloprovincialis*	gills
PRJNA525609	SRR8713540	*Mytilus galloprovincialis*	mantle
PRJNA525609	SRR8713541	*Mytilus galloprovincialis*	mantle
PRJNA525609	SRR8713542	*Mytilus galloprovincialis*	mantle
PRJNA525609	SRR8713543	*Mytilus galloprovincialis*	mantle
PRJNA525609	SRR8713544	*Mytilus galloprovincialis*	mantle
PRJNA525609	SRR8713545	*Mytilus galloprovincialis*	mantle
PRJNA525609	SRR8713546	*Mytilus galloprovincialis*	mantle
PRJNA525609	SRR8713547	*Mytilus galloprovincialis*	mantle
PRJNA486919	SRR7725723	*Mytilus galloprovincialis*	gills
PRJNA470760	SRR7145588	*Mytilus galloprovincialis*	hemocytes
PRJNA470760	SRR7145589	*Mytilus galloprovincialis*	hemocytes
PRJNA470760	SRR7145590	*Mytilus galloprovincialis*	hemocytes
PRJNA407401	SRR6041063	*Mytilus galloprovincialis*	digestive gland
PRJNA407401	SRR6041064	*Mytilus galloprovincialis*	digestive gland
PRJNA407401	SRR6041065	*Mytilus galloprovincialis*	digestive gland
PRJNA407401	SRR6041066	*Mytilus galloprovincialis*	digestive gland
PRJNA326100	SRR6238453	*Mytilus galloprovincialis*	digestive gland
PRJNA326100	SRR6238454	*Mytilus galloprovincialis*	digestive gland
PRJNA326100	SRR6238455	*Mytilus galloprovincialis*	digestive gland
PRJNA326100	SRR6238446	*Mytilus galloprovincialis*	digestive gland
PRJNA326100	SRR6238447	*Mytilus galloprovincialis*	digestive gland

Table S2. RNAseq raw-data (NCBI Sequence Read Archive database) found for *P. lividus and S. purpuratus*

Bioproject	SRA accession	Species	Tissue
PRJNA625933	SRR11561266	*Paracentrotus lividus*	testis
PRJNA625934	SRR11561267	*Paracentrotus lividus*	testis
PRJNA625935	SRR11561268	*Paracentrotus lividus*	testis
PRJNA625936	SRR11561269	*Paracentrotus lividus*	ovary
PRJNA625937	SRR11561270	*Paracentrotus lividus*	ovary
PRJNA625938	SRR11561271	*Paracentrotus lividus*	ovary
PRJNA264358	SRR1664663	*Paracentrotus lividus*	0hpf*
PRJNA264358	SRR1735496	*Paracentrotus lividus*	4hpf*
PRJNA264358	SRR1735498	*Paracentrotus lividus*	18hpf*
PRJNA264358	SRR1735500	*Paracentrotus lividus*	24hpf*
PRJNA264358	SRR1735501	*Paracentrotus lividus*	48hpf*
PRJNA81157	SRR531953	*Strongylocentrotus purpuratus*	coelomocytes
PRJNA81157	SRR531955	*Strongylocentrotus purpuratus*	gut
PRJNA81157	SRR531958	*Strongylocentrotus purpuratus*	ovary
PRJNA81157	SRR532121	*Strongylocentrotus purpuratus*	testis
PRJNA531297	SRR8863032	*Strongylocentrotus purpuratus*	unfertilized eggs

*used only for the de novo transcriptome assembly
